# Simple and Label-Free Fluorescent Detection of Melamine Based on Melamine–Thymine Recognition

**DOI:** 10.3390/s18092968

**Published:** 2018-09-06

**Authors:** Hualin Yang, Jiujun Wang, Qinghua Wu, Yun Wang, Li Li, Baomiao Ding

**Affiliations:** 1 Edible and Medicinal Fungi Research Center, Yangtze University, Jingzhou 434025, China; yanghualin2005@126.com (H.Y.); 1wangyun@yangtzeu.edu.cn (Y.W.); 2 College of Life Science, Yangtze University, Jingzhou 434025, China; wangjiujun2018@126.com (J.W.); wqh212@hotmail.com (Q.W.); lily2012@yangtzeu.edu.cn (L.L.)

**Keywords:** melamine detection, melamine-thymine recognition, SYBR Green I, exonuclease I

## Abstract

In the past few years, melamine has been illegally added into dairy products to increase the apparent crude protein levels. If humans or animals drink the milk adulteration of melamine, it can form insoluble melamine–cyanurate crystals in their kidneys which causes kidney damage or even death. In the present work, we constructed a simple and label-free fluorescent method for melamine detection based on melamine-thymine recognition. SYBR Green I was utilized as a reporter for this method as it did not require any modification or expensive equipment. In the absence of melamine, polythymine DNA was digested by Exo I, which caused a decrease in the fluorescence signal. In the presence of melamine, the polythymine DNA was able to fold into a double chain structure, however this was done with the help of T-melamine-T mismatches to prevent degradation. Then, the SYBR Green I combined with the double-stranded DNA to result in an intense fluorescence signal. The limit of detection in this method was 1.58 μM, which satisfied the FDA standards. This method also had a good linear relationship within the range of 10–200 μM. In addition, this new method has a good selectivity to distinguish melamine from the component of milk. As a result, we developed a simple and highly selectivity method for melamine detection.

## 1. Introduction

Melamine (2,4,6-triamino-1,3,5-triazine) is a small organic compound widely used in the production of plastics, fertilizer, resins and other products [1,2]. In the past few years, melamine has been illegally added into dairy products to increase the apparent crude protein levels. This is because the standard method for protein level in food is the Kjeldahl method, which is done by measuring nitrogen content, and the nitrogen content of melamine (66% by mass) is much higher than protein (~16%). If humans or animals drink the milk adulteration of melamine, it can form insoluble melamine–cyanurate crystals in their kidneys which causes kidney damage or even death [3,4,5]. In 2008, melamine caused several infant deaths and thousands of people had kidney failures in China [6,7]. As a result, many countries have formulated regulations on the maximum content limits of melamine in food, especially milk products. The Federal Food and Drug Administration (FDA) has defined a limitation of melamine as 2.5 ppm (19.8 μM) for non-infant products and 1 ppm (7.9 μM) for infant milk products [8,9]. Therefore, sensitive and selective detection melamine is very important for people’s health.

Thus far, many methods and techniques have been designed to detect melamine [10]. Traditional melamine detection methods include high performance liquid chromatography (HPLC) [11], gas chromatography-mass spectrometry (GC-MS) [12], liquid chromatography-mass spectrometry (LC-MS) [13] and capillary electrophoresis (CE) [14]. These instrumental analytical methods can achieve high sensitivity and selectivity. However, expensive instrumentation, skilled manpower, and complex sample pretreatment are usually required, which greatly limit their utility in routine melamine determination. Recently, a number of detection methods have been developed to detect melamine based on gold nanoparticles [15,16,17,18], electrochemical signal [19,20], and immunoassay approaches [21,22] to overcome the above-mentioned problems. However, synthesis or modification of gold nanoparticles and electrode are often tedious and costly. Moreover, melamine being a semi-antigenic substance makes preparation of specific antibodies a difficult process. Thus, it is essential to design a simple, convenient and sensitive strategy for melamine determination.

Recently, some papers have reported that melamine can form T-melamine-T mismatches in oligonucleotides by triple hydrogen bonding with thymine base [23,24,25,26]. Furthermore, SYBR Green I can exhibit a dramatic enhancement upon binding to double-stranded DNA [27,28]. In addition, Exonuclease I (Exo I) is a single strand exonuclease which catalyzes the removal of nucleotides from single-stranded DNA in the 3′ to 5′ direction [29,30]. Inspired by these reports, we proposed a simple and label-free fluorescent detection of melamine that could be developed by the combination of T-melamine-T mismatches, SYBR Green I and Exo I.

## 2. Materials and Methods

### 2.1. Materials

The synthetic oligonucleotides DNA purified by PAGE was obtained from Shanghai Sangon Biological Engineering Technology & Services Co., Ltd. (Shanghai, China). SYBR Green I was purchased from Solarbio Science & Technology Co., Ltd. (Beijing, China). Melamine, lactose, glucose, sucrose, CaCl_2_, CuCl_2_, FeCl_3_, KCl, MgCl_2_, MnCl_2_, vitamin B2 and vitamin C were purchased from Sinopharm Chemical Reagent Co., Ltd. (Beijing, China). Exonuclease I was purchased from Takara Biotechnology Co. Ltd. (Dalian, China). All chemicals were analytical reagent. The water used was purified by Millipore (Burlington, MA, USA) Milli-Q (18 MΩ/cm). Stock solution of oligonucleotides (100 μM) was prepared by deionized water. Before used, the oligonucleotides solution was diluted to the required concentration with deionized water.

### 2.2. Fluorometric Analysis

All fluorescence measurements were performed on a F-7000 spectrometer (Hitachi, Tokyo, Japan). The instrument settings were as follows: λ_EX_ = 495 nm (bandpass 5 nm), λ_EM_ from 505 nm to 700 nm (bandpass 5 nm) and the photomultiplier tube (PMT) detector voltage = 500 V.

The sensitivity of this method was performed as follows. First, different concentrations of melamine were added into 1× Exo I buffer containing 500 nM polythymine DNA. Then, the mixture was incubated at 37 °C for 2 h to ensure that DNA and melamine were fully bind. Second, 2.5 U Exo I was added and incubated at 37 °C for 0.5 h, then it was heated up to 80 °C for 15 min to deactivate Exo I. Finally, 1× SYBR Green I was added and incubated at room temperature for 20 min, before the fluorescence measurements were taken. The fluorescence signal change was calculated with the formula Y = F/F_0_, where F_0_ and F were the area under curve of the emission spectra in the absence and presence of melamine, respectively.

The selectivity of this method was performed using the same procedure as in the sensitivity test, in the presence 40 μM melamine and 400 μM interfering materials.

### 2.3. Detection of Melamine in Milk Sample

Raw milk samples were pretreated before detection. First, 5 mL of raw milk was placed into a 50 mL centrifuge tube and 15 mL trichloroacetic acid was added. Second, the solution was mixed with a vortex for 10 min and centrifuged at 4500 r/min for 10 min to deposit protein. Third, the supernatant was collected and adjusted to pH 7.0 by 6 M NaOH. Finally, the supernatant was filtered into a new centrifuge tube through a 0.22 µm membrane. The obtained solution was used for detection according to the method in Section 2.2.

## 3. Results

### 3.1. Detection Principle

**Figure 1:**
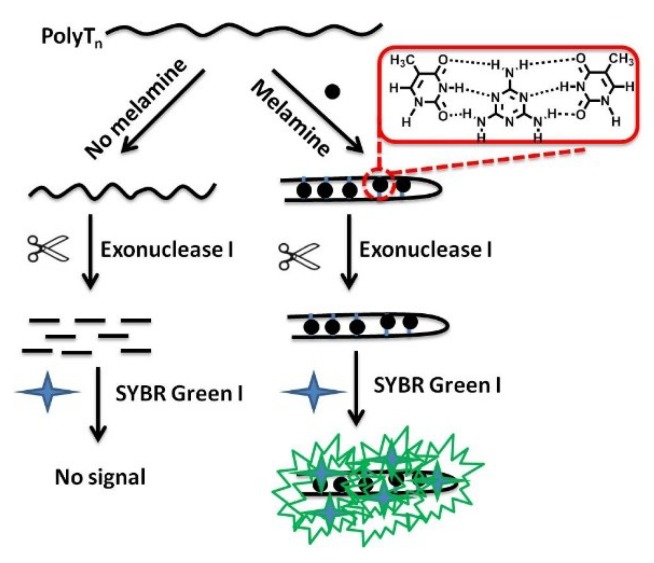
Schematic illustration of the simple and label-free fluorescent method for melamine based on melamine–thymine recognition.

The principle of the proposed strategy for melamine detection was illustrated in Figure 1. First, polythymine DNA was designed. In the absence of melamine, polythymine DNA was digested by Exo I because it existed as a single strand of DNA. When the SYBR Green I was added, no fluorescent signals were detected. In the presence of melamine, the polythymine DNA could fold into a double chain structure, however T-melamine-T mismatches was used prevent degradation. Then, the SYBR Green I combined with the double-stranded DNA to result in an intense fluorescence signal.

### 3.2. Optimize the Length of Polythymine DNA

**Figure 2:**
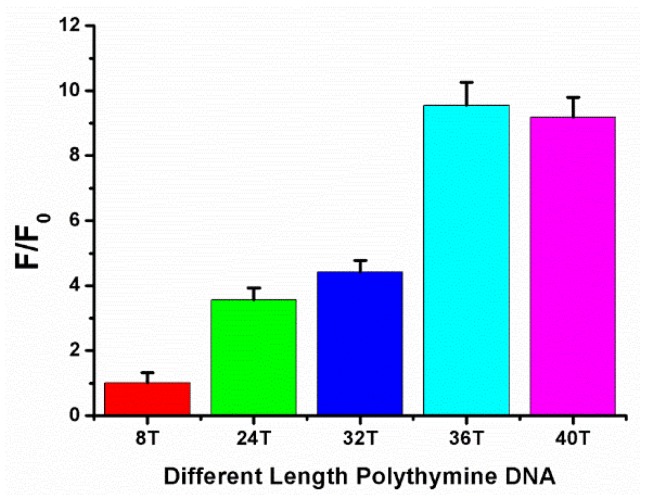
The fluorescence signal change (F/F_0_) under different length polythmine DNA. where F_0_ and F were the area under curve of the emission spectra in the absence and presence of 500 μM melamine, respectively. Error bars were estimated from at least three independent measurements.

In the first place, different length polythymine DNA (polyT_8_, polyT_24_, polyT_32_, polyT_36_, polyT_40_) were initially screened by comparison the fluorescence signal change (F/F_0_) (Figure 2). F and F_0_ were the area under curve of the emission spectra in the presence and absence of melamine, respectively. This signal change gradually increased with the length of the DNA up to 36 T. Then, F/F_0_ had a slight fall as the DNA length increased to 40 T. So, polyT_36_ was selected so as to guarantee better effect of measurement and save some cost.

### 3.3. The Sensitivity of Our Proposed Strategy

**Figure 3:**
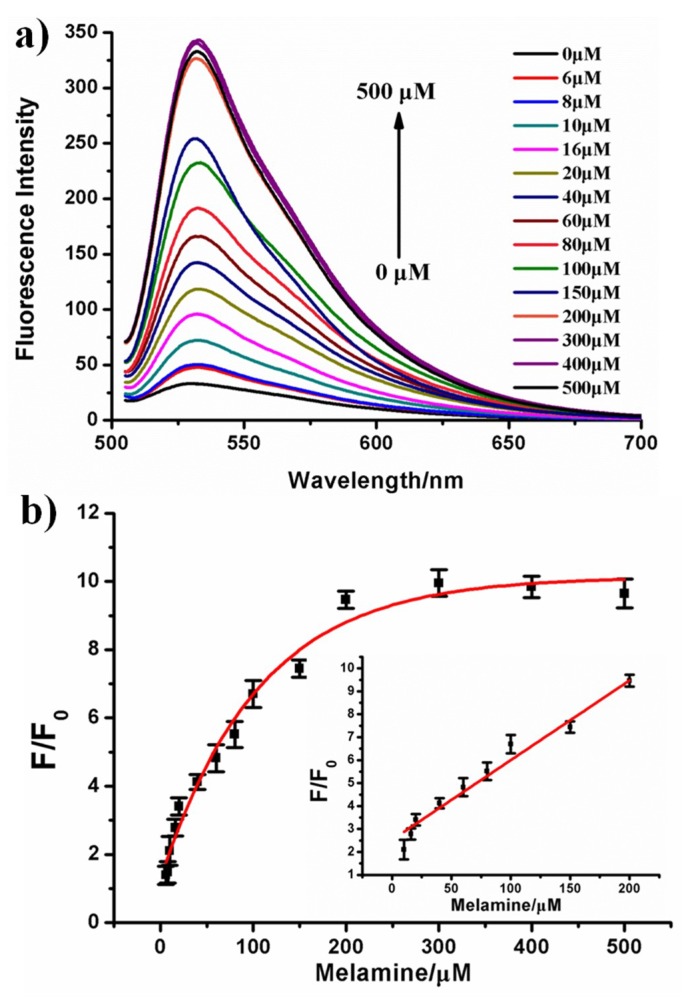
(**a**) The fluorescence emission spectra in the presence of difference melamine concentrations. (**b**) The fluorescence intensity signal change F/F_0_ for melamine concentrations ranging from 6 to 500 μM, which matched the dose-response curve. Inset: the signal change matched a linear relationship at concentrations of 10–200 μM. F_0_ and F are the area under curve of the emission spectra in the absence and presence of melamine, respectively. Error bars were estimated from at least three independent measurements.

Subsequently, the sensitivity of the proposed strategy was measured using different concentrations of melamine. As shown in Figure 3a, with the increase in concentration of melamine, the fluorescence signal intensity also gradually increased. Even at 6 μM melamine concentration, the fluorescence signal change was still clearly observed to increase from 32.7 to 45.6. This concentration (6 μM) was lower than the maximum safety limit in infant milk products (7.9 μM) set by the FDA. With the melamine concentration increased to 200 μM, the fluorescence signal had a 9.5-fold increase (from 32.7 to 309.8). This result indicated that double stranded DNA structure was highly associated with the concentration of melamine. Then, the signal change (F/F_0_) were studied in the presence of varying melamine concentrations (Figure 3b). The results showed that the calibration curve matched the dose-response curve (R^2^ = 0.976). In addition, the signal change (F/F_0_) exhibited a linear correlation within the melamine concentration range from 10 to 200 μM. The regression equation was Y = 2.5308 + 0.0347X (R^2^ = 0.978), where Y and X represented the signal change (F/F_0_) and the concentration of melamine, respectively (inset in Figure 3b). The limit of detection (LOD) was estimated to be 1.58 μM, this was based on 3 times the standard deviation of a blank response. Such a limit was lower than the maximum safety limit set by the FDA for non-infant-formula products (19.8 μM).

### 3.4. The Selectivity of Our Proposed Strategy

**Figure 4:**
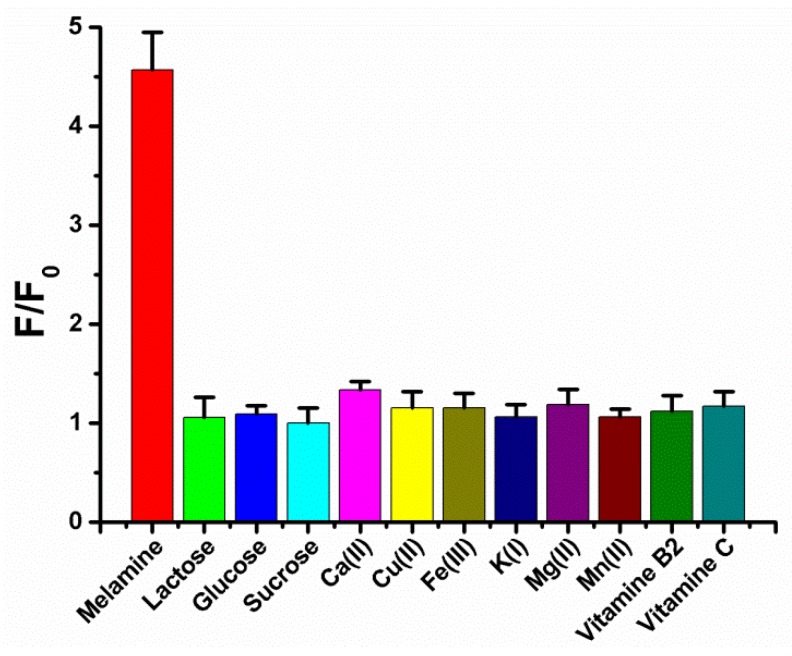
The selectivity of our method was evaluated in the presence 40 μM melamine and 400 μM interfering materials. Error bars are estimated from at least three independent measurements.

Additionally, the interference of the component of milk was an important factor used to consider if the new method can be used to determine melamine in whole milk. The selectivity of our proposed strategy was evaluated in the presence of lactose, glucose, sucrose, Ca^2+^, Cu^2+,^ Fe^3+^, K^+^, Mg^2+^, Mn^2+^, vitamin B2 and vitamin C. The result in Figure 4 showed that melamine exhibited the highest signal change (F/F_0_) and interfering materials were almost negligible even if the concentrations were 10 times higher than melamine. We conclude that there was only weak affinity between interfering materials and polythymine DNA by few or no hydrogen bond formation. These results show that the strategy had a good selectivity towards melamine. This result was also consistent with other reported methods [16,23] which was based on melamine–thymine recognition to detect melamine.

### 3.5. The Real Sample Analysis in Milk Samples

With the ideal sensitivity and selectivity, the applicability of this method to real milk samples was examined. Because the melamine-milk sample was not obtained in hand, we took the spiked milk to evaluate the reliability as other papers [15,16,18,26]. Standard addition method was used on three standard milk samples and the final melamine concentrations were obtained as 20, 80, 150 μM. After the pretreatment of milk samples, the concentrations of melamine were detected using the proposed method. The experimental results are shown in Table 1 and the recovery of our method ranged in between 99.17–109.75%. These results clearly revealed that the proposed approach might have a potential practical application to measure the melamine in raw milk samples.

## 4. Conclusions

In the present work, we developed a simple and label-free fluorescent method for melamine detection based on melamine-thymine recognition. SYBR Green I was utilized as a reporter for this method as it did not require any modification or expensive equipment. The limit of detection in our method was 1.58 μM, which satisfied the FDA standards. In addition, this method had a good selectivity to distinguish melamine from the component of milk. As a result, the method has great potential to be applied in bioanalytical researches and food safety detection.

## Figures and Tables

**Table 1:** Determination of melamine in milk samples using the proposed method.

Sample Number	Melamine Added (μM)	Melamine Detected (μM)	Recovery (%)
1	20	21.95 ± 2.21	109.75
2	80	86.12 ± 5.09	107.65
3	150	148.76 ± 8.43	99.17
